# A review of canola meal as an alternative feed ingredient for ducks

**DOI:** 10.1186/s40781-015-0062-4

**Published:** 2015-09-01

**Authors:** Samiru Sudharaka Wickramasuriya, Young-Joo Yi, Jaehong Yoo, Nam Kyu Kang, Jung Min Heo

**Affiliations:** Division of Animal and Dairy Science, Chungnam National University, Daejeon, 305-764 Republic of Korea; Division of Biotechnology, College of Environmental & Bioresources, Chonbuk National University, Iksan-si, Jeollabuk-do 570-752 Republic of Korea

**Keywords:** Anti-nutritional factors, Canola meal, Ducks, Feeds

## Abstract

This review provides an overview of the published data on the canola meal and its suitability for duck as an alternative plant-origin protein source to soybean meal. Canola meal is a legume origin protein source containing comparable amino acid profile to soybean meal and rich in essential minerals and vitamins. Nonetheless, it is known to contain less in energy content than soybean meal. Factors like field conditions and processing methods creates compositional variations among canola meal. Presence of anti-nutritional factors such as phenolic substances, phytate and glucosinolates which are known to reduce growth performance in livestock animals, are the major drawbacks for canola meal to be a competitive plant-origin protein source in the feed industry. This review is focused to address i) nutritional characteristics and feeding value of canola meal for ducks and ii) impacts of feeding canola meal on performances of ducks.

## Introduction

Due to increasing diet cost in animal production industry, it has been becoming important to explore alternative feed ingredients for cost effective animal production. The use of less expensive protein and amino acid source is one of the ways to reduce the feed costs in the area of animal production. Concurrently, the effect of feed ingredients on growth performance of host animals should be carefully accounted for proper evaluation of an alternative ingredient [1].

Soybean meal has long been used as a reference plant-origin protein source in the animal feed industry. However, the cost of using soybean meal can increase cost of diet, and in turn many poultry producers are searching for alternative sources of supplementary protein source that are cost effective [2, 3].

Canola is a new variety of rapeseed which developed using plant breeding techniques to reduce the toxic glucosinolate content [4]. Canola meal is an oil seed meal and contains high concentration of protein and a well-balanced amino acid profile [5]. In addition, essential minerals and vitamins, such as choline, biotin, folic acid, niacin, riboflavin and thiamin are available in canola meal [6–8]. Nonetheless with compared to soybean meal, inclusion of canola meal in diets for birds have been limited due to less-protein, less-energy and higher dietary fiber [6, 9]. The development of low erucic acid and low glucosinolate cultivars of canola has resulted in increased usage of canola meal in poultry rations [10, 11]. Only a few studies demonstrated that duck utilize energy in canola meal more efficiently than chickens [11–14], however comparable amino acid digestibility was found in both ducks and chickens [15].

Rearing ducks for the production purpose (i.e., eggs and meat) are an advantageous activity in livestock industry as they are rather easier and cheaper to grow than chickens. Utilization of canola meal as an alternative plant protein source for duck is having a great value in terms of cost minimization [16].

A couple of review articles [6, 17] have attempted to elaborate factors effecting nutritive value of canola meal. Nonetheless, canola meal on ducks was not focused nor evaluated. Further, published data pertaining to the nutritive values of canola meal for ducks are limited. This review, therefore, will be focused on i) nutritional characteristics and feeding value of canola meal for ducks, and ii) impact of feeding canola meal on performances of ducks.

## Review

### Duck production

Duck production is very popular and has a great demand in many parts of the world [18]. Meat products of ducks were the third highest poultry meat production [19]. Duck meat production was expanded by 1.3 million tons from 2000 to 2011 in worldwide and yet, the trend is continuing. Asia is the leading duck producing region compare to other regions [20]. With compared to chicken meat, duck meat is having distinctive characteristics as a red meat [18]. With the increasing demand many duck meat retail cuts become more available for the diet conscious customers [18]. Beside from the duck meat, egg and down feathers are another two main products that have great demand [21].

Profitability of the duck production can be increased by adapting the unconventional feed to the diet formulation which reduces the production cost, especially by utilizing low cost plant protein over animal protein [16].

## Chemical and nutritional composition

### Proximate composition

Canola meal is considered as a source of vegetable protein for livestock industry. Considered principal nutrient components of canola meal include protein, carbohydrates, crude fiber and ash along with residual oil which is not removed through the oil extraction process (Table 1). Although canola meal commonly used as a protein source in animal diets, it contains lower protein (36 %), and available energy (2,000 kcal/kg AME) but higher fiber (12 %) content compared with soybean meal (7 %) [22].

**Table 1:** Chemical composition of canola meal (as fed basis)

Component	Solvent form	Expeller form	Reference
Dry matter, %	88.0	82.9	[23]
Crude protein, %	37.3	36.3	[24]
	36.0	36.3	[23]
	37.3	34.5	[15]
	34.0	32.0	[25]
	–	36.3	[26]
	34.0	–	[27]
Crude fiber, %	9.85	10.6	[24]
	12.0	10.6	[23]
	15.0	14.0	[25]
	–	6.9	[26]
	9.3	–	[27]
Ash, %	7.3	6.3	[24]
	6.1	6.3	[23]
	–	6.9	[26]
	6.8	–	[27]
Fat, %	3.4	11.1	[24]
	3.5	11.1	[23]
	3.5		[27]
Moisture, %	10.7	7.1	[24]
	12	11	[25]
	–	5.6	[26]
	11.1	–	[27]

Dashes indicate that no data were available

Proximate composition of canola meal varies due to cultivars, environment conditions during growing and harvesting periods and crushing conditions [28]. For example, dry seasonal conditions create lower oil content and higher level of protein content [24]. Furthermore, canola meal quality is influenced by the type of oil extraction process (i.e., expeller- and solvent-extraction) [29]. Level of heating duration contributes to degradation of heat sensitive amino acids such as lysine [23]. Expeller-pressed canola meal contains more residual oils than solvent-extracted canola meal [24, 30].

Dietary protein content in canola meal can be highly influenced by the protein content of canola seeds [24]. Additionally, it relates with carbohydrate fraction, oil fractions along with the oil extraction efficiency from seed and changes in moisture content during the processing [24]. According to Canadian Canola Council, the minimum crude protein guarantee for Canadian canola meal is 36 % (8.5 % moisture basis). However, actual protein content is usually 36–39 % (Table 1). It depends on variation in canola seed composition yearly due to growing and harvest conditions [23].

Although protein content depends on growing conditions of canola, protein quality is directly related with processing method [31, 32]. In particular, reduction in protein digestibility and protein quality are influenced by heat treatments, use for oil extraction and subsequent desolventizing and toasting of canola seed [32]. In addition, some [33, 34] demonstrated that soluble protein content decreased during the heat-treatment, and also resulted in lowering protein depositon, ileal-digestible lysine retention and growth performance of host animals. Protein quality of canola meal for ducks is scantly documented and aforementioned findings are based on other monogastrics.

Canola meal also contains complex carbohydrates fractions [23] (Table 2). Fiber components of canola meal include lignin with associated polyphenols (8 %), cellulose (4–6 %) and non-cellulosic polysaccharides (13–16 %) which consist predominantly of pectic substances [35]. Other important components include oligosaccharides (2.5 %), glycoprotein (5 %; i.e., arabinogalactan-protein, cell wall protein), phytate (3.3 %), minerals associated with the fiber fraction (1 %) and gums (4 %). Similar to other nutrients, variability may be caused by several factors including method of analysis, genetic differences, harvest management and environmental conditions during the growing stage [6]. For example, yellow-seed *Brassica campestris* had a lower percentage seed coat (hull) than brown seed types [36]. Although yellow hulls evident that lower fiber content, yellow seed embryos contains high measures of fiber over the brown seeds [6].

**Table 2:** Carbohydrate components of canola meal

Component	Value (g/kg)				
	[6]^a^	[23]^b^	[17]^c^	[25]^c^	[37]^b^
Starch	25	51	24	52	–
Sugars	–	67	-	80	–
Sucrose	77	62	60	–	–
Fructose + glucose	–	05	–	–	–
Cellulose	49	45	–	46	46
Oligosaccharides	25	22	20	23	23
Non-starch polysaccharides (NSP)	179	157	180	161	161
Soluble NSP	15	14	–	14	14
Insoluble NSP	164	144	-	147	147
Crude fiber	146	117	116	120	120
Acid detergent fiber	198	168	182	172	172
Acid detergent lignin	–	51	–	–	
Neutral detergent lignin	–	207	–	–	
Total dietary fiber	331	323	317	330	330

Dashes indicate that no data were available

^a^Oil-free, dry matter

^b^12 % moisture basis

^c^10 % moisture basis

### Nutritional content and minerals

Canola meal is considered as a quality source of essential minerals (Table 3). In addition, some documented that it is a relatively good source of essential minerals compared to other vegetable-origin oilseeds [23]. Similarly, a couple of studies [1, 38] determined that canola meal contains higher calcium and phosphorus contents than soybean meal. Although 65 % of the phosphorus in canola meal is in the phytate form which is not available for monogastric animals, it is still a better source of calcium and phosphorus compared to soybean meal [1, 38]. Documented data pertaining to mineral availability of canola meal on duck were scare. However, feeding canola meal along with in-feed phytase would improve availability of minerals for monogastric animals [39]. In addition, canola meal appears to be a quility source of selenium relative to other oilseed meals [37].

**Table 3:** Mineral composition of the canola meal and soybean meal

Mineral	Canola meal				Soybean meal	
	[24]^a^	[28]^b^	[17]^c^	[40]^d^	[17]^c^	[40]^d^
Calcium, %	0.56	0.70	0.67	0.68	0.33	0.27
Phosphorus, %	0.96	1.13	1.02	1.17	0.66	0.62
Phytate phosphorus, %	0.83	–	0.64	–	0.38	–
Sodium, %	–	–	0.08	–	0.01	–
Chlorine, %	0.10	–	0.10	–	0.05	0.05
Potassium, %	1.26	1.35	1.17	1.29	2.00	–
Sulphur, %	0.62	0.94	0.65	–	0.44	–
Magnesium, %	0.47	0.57	0.56	0.64	0.28	–
Copper, mg/kg	3.9	6.34	–	10	–	15
Iron, mg/kg	138	157	–	159	–	170
Manganese, mg/kg	52	54.7	–	54	–	43
Molybdenum, mg/kg	-	1.5	–	-	–	–
Zinc, mg/kg	45	57.8	–	71	–	55
Selenium, mg/kg	–	1.22	–	1.00	–	0.1

Dashes indicate that no data were available

^a^As fed basis (*n* = 26)

^b^Dry matter basis (*n* = 28)

^c^10 % moisture basis

^d^As fed basis

In terms of vitamin content, canola meal has a greater vitamin compared to soybean meal (Table 4) but there is limited information available on vitamins composition of canola meal. It was reported that canola meal contains greater amounts of B vitamins such as biotin, folic acid, niacin, riboflavin and thiamin compared with soybean meal [4].

**Table 4:** Vitamin content of the canola meal and soybean meal (As fed basis)

Vitamins (mg/kg)	Canola meal				Soybean meal		
	[37]	[6]	[23]	[41]	[40]	[16]	[42]
Vitamin E (alpha tocopherol)^a^	20.89	21.64	13	–	4.47	3.58	3.43
Pantothenic acid	9.5	9.5	9.3	9.5	15	16.3	15
Niacin	160	160	156	160	22	28	22
Choline	6700	6700	6500	6700	2700	2609	2730
Riboflavin	5.8	5.8	5.7	5.8	2.9	2.9	3.1
Biotin	1.1	1.07	0.96	0.98	0.32	0.32	0.26
Folic acid	2.3	2.3	0.8	0.83	1.3	0.6	1.37
Pyridoxine	7.2	7.2	7.0	-	5.0	6.0	-
Thiamin	5.2	5.2	5.1	5.2	3.2	6.0	3.2

Dashes indicate that no data were available

^a^Units in IU/Kg

Canola meal has a reasonable amount of amino acid (Table 5) containing high amount of sulfur amino acid, [43, 44] while comparatively lower in lysine and arginine-content than soybean meal [17]. Prepress solvent extracted canola meal is characterized with lower and less consistent amino acid digestibility in broilers than soybean meal [43, 40]. Some amino acids, especially lysine can be turned to biologically unavailable lysine derivatives (un-reactive lysine) during heat processing as well as prolonged storage of feedstuffs [45, 46].

**Table 5:** Amino acid content (As fed basis)

Amino acid (g/kg)	Expeller extracted canola meal			Solvent extracted canola meal				Soybean meal	
	[24]	[26]	[5]	[24]	[27]	[28]^a^	[5]	[47]	[25]
Methionine	7.0	7.1	7.0	7.2	7.1	8.6	7.2	6.6	6.3
Cystine	8.6	–	8.5	8.7	–	12.0	8.6	6.6	7.3
Methionine + Cystine	15.6	–	15.6	16.0	–	–	15.9	–	13.6
Lysine	19.7	19.8	19.6	20.2	20.2	24.9	20.0	30.4	28.9
Threonine	15.0	15.1	15.0	15.6	15.6	19.0	15.7	18.2	18.4
Tryptophan	4.9	4.4	4.8	5.1	3.9	5.3	5.0	6.8	6.3
Arginine	21.5	21.5	21.0	22.1	21.8	26.0	21.8	35.6	34.8
Isoleucine	13.9	15.0	13.9	14.3	13.9	17.8	14.3	22.4	21.7
Leucine	24.3	25.2	24.3	25.3	25.5	30.4	25.4	37.6	36.0
Valine	17.9	18.8	17.9	18.6	18.0	22.9	18.6	23.6	23.0
Histidine	9.5	9.6	9.5	9.9	9.6	15.4	9.8	12.5	12.1
Phenylalanine	14.1	14.3	13.9	14.6	14.6	17.1	14.6	24.3	23.7

Dashes indicate that no data were available

^a^Ether extracted dry matter basis

The fatty acid profile is different between expeller and solvent extracted meal. Palmitic acid and linoleic acid were consistently higher in solvent extracted meal but oleic and linolenic acids were lower than expeller meal [24]. This remarks mechanical extraction leads to superior removal of oleic and linolenic acids from the seed [24].

###  Anti-nutritional factors in canola meal

Canola meal cannot be substantial as full protein supplement in poultry diet, due to the inherent anti-nutritional factors (ANF) (Table 6). These ANF hinder animal growth performance by interfering with nutrient absorption in the digestive system [44]. Phenolics are one of the ANF which is unwanted and undesirable in animal feed. Among all phenolics, sinapine is the most abundant phenolic in canola that caused ‘fishy’ or ‘crabby’ tainted eggs in hens [44]. However, off flavor has not been detected in broiler carcasses due to the sinapine [17]. To eliminate sinapine from meal, plant breeding is economically better than physical process [48]. Another ANF found in canola meal is phytate, a complex of inositol and phosphorus [49]. Phytate is not bioavailable as a phosphorus source to non-ruminant animals and fish due to lack of enzyme degrading phytate phosphorus [44]. Feeding phytate in non-ruminant animals can lead to a hypertrophy of the thyroid glands, and subsequently to lower growth performance [50–52]. Hydrolyzing phytic acid by enzymatic methods (exogenous phytase) was studied previously [53, 54], and they found that about 72–73 % of meal phytate could be hydrolyzed by addition of phytase [54] and improved feed to gain ratio. Whilst, no significant growth performance was observed [54]. It can be applied to canola meal to overcome above mentioned potential problems. Canola meal contains considerable amounts of glucosinolates that can break down into various compounds (i.e., thiocyanate, isothiocyanate, oxazolidinethione and nitriles) which are known to have a negative effect upon animal growth performance. Level of glucosinolates found in canola seed ranges from 3.6 to 9.2 μmol/g [24] and this content should restrict to 1–1.5 μmol/g feed and to even lower concentrations for young monogastric animals [55]. However, this level can be up to 4 μmol/g for layers and 1.5 μmol/g was recommended for broilers [17].

**Table 6:** Amount of anti-nutritional factors in canola meal and their main effects

Component				References
Sinapine %	Phytic acid %	Glucosinolates (μmol g^−1^)	Tannins, %	
*Average amount*				
0.6–1.8	3.0–6.0	18.3	1.5–3.0	[37]
0.6–1.8	3.0–6.0	10–12	1.5–3.0	[6]
1.0	4.0	16	1.5	[25]
1.0	–	5.5	–	[17]
0.79–0.97	–	1.73–5.26	–	[5]
*Effects on poultry*				
Bitter flavor.	Binds minerals	Enlargement of internal organs like liver and Kidney	Impairs digestion. Especially protein	[6]
Layers produce “Fishy eggs”.				
Production of off flavor “Fishy eggs”	Render the minerals and make them unavailable for absorption	Decrease the growth rates of broilers, increase the thyroids and liver sizes and cause hemorrhagic liver syndrome	Interfere with digestive enzymes and lower the nutrient utilization	[37]
By layer hens				
–	–	No real evidence that canola cultivars with zero glucosinolates may cause any effect	–	[25]
“Fishy taint” in brown shell egg	Make protein and minerals biologically unavailable.	Interfere with the function of thyroid gland and adversely affect growth performance	Affect protein digestion and poor growth performance in broiler	[17]

Dashes indicate that no data were available

High fiber content of canola meal is one of the critical factors limiting the increased use of canola meal in poultry diets [56–59]. Dietary fiber contents account for approximately one-third of the meal, and consist of cellulose (4–6 %), non-cellulosic polysaccharides (13–16 %), lignin and polyphenols (5–8 %), and protein and minerals associated with the fiber fraction [35]. Large amounts of crude fiber are present in the hulls of oil seeds, which contain carbohydrate fraction and proteins along with non-starch polysaccharides (NSP) [60]. Dehulling is one of the methods to reduce negative effects of the fiber. It has been documented that dehulling improved digestibility (for amino acid), amino acid and energy utilization of canola meal when fed to broiler chicken [61]. However, dehulling increases the unit cost of production in the commercial scale [62]. The simple way to overcome this problem is through genetic selection and plant breeding to make the seed coat thinner. This provides alternative way to improve the canola meal quality. It may be possible to breed a new variety of canola with thin seed coat, lower fiber and more protein contents [63, 64]. A recent study revealed superior quality characters in canola meal derived from the newly developed low fiber fraction line of yellow-seeded *B. napus* with having more protein, more sucrose, and less dietary fiber contrasting to black-seeded *B. juncea* [22]*.*

### Energy content

The energy content of canola meal was evaluated for poultry last for 20 years (Table 7). The amount of energy supply from canola meal is directly related to the residual oil in the meal. However, canola meal consists with comparatively lower metabolizable energy (ME) level than other protein sources such as soybean meal in poultry diet [43, 40]. This is because of the higher fiber content of the canola meal that dilutes the energy content [61]. The ME content of expeller-pressed canola meal is higher than that of solvent-extracted canola meal due to higher residual oil [65]. Metabolizable energy values of the meal have improved via plant breeding by reducing glucosinolate levels [6]. Dehulling of canola meal resulted in improved ME levels along with digestible energy value. Canola meal contains about 3,346 kcal/kg digestible energy while dehulled canola meal contains approximately 4,063 kcal/kg digestible energy, due to reduction of fiber component in hulls which comprise about 12–16 % of canola seeds [6]. Other factors that may influence the ME content of meal include its content of fiber, protein and oil. These factors are influenced by variety and seed quality along with feed processing technology [6]. Additivity and associative effects of metabolizable energy in canola meal was studied for white Pekin ducks [14]. It was reported 2,186 kcal/kg AMEn and 2,439 kcal/kg TMEn values for canola meal, which are higher (2,000 and 2,070 kcal/kg, respectively) than NRC (1994) values. Moreover, those energy values are higher in ducks than chickens [14].

**Table 7:** Available energy value for canola meal (kcal/kg)^a^

AMEn	TME	TMEn	Reference
1980	–	2090	[13]^b^
2000	–	2070	[23, 40]^c^
2390	–	–	[23]^c^
2186	2764	2439	[14]^d^
–	2049	1964	[66]^b^
2390	–	–	[67]^e^
1910	–	–	[67]^c^

Dashes indicate that no data were available

^a^Abbreviations are *AMEn* Apparent metabolizable energy, N-corrected, *TME* True metabolizable energy and *TMEn* True metabolizable energy, N-corrected

Animal used : ^b^White Leghorn roosters, ^c^Broilers, ^d^White Pekin ducks, ^e^Layer hen

### Amino acid for ducks

With regard to the amino acid requirement of ducks, greatest attention should be given to the sulfur amino acids (i.e., methionine and cysteine) because they are generally considered to be the first limiting amino acids for ducks [42] similar to other poultry [68]. Requirement of total sulfur amino acids for male Muscovy ducklings was 0.60 % (diet contain, 988 kcal/kg ME) and 0.55 % (diet contain 3,090 kcal/kg ME) to achieve maximal growth from 3 to 6 and 6 to 10 weeks, respectively [69]. Based on straight-run Mule ducklings, it was concluded that 0.59 % total sulfur amino acids were required (diet contain 2,892 kcal/kg ME) for maximal growth and feed efficiency [70].

Canola has a reasonably well balanced amino acid profile for ducks. Although it is deficient in lysine content, it is rich in sulfur amino acids [17]. A recent study confirmed methionine and cysteine contents were higher in canola meal (0.91 %: 1.21 %) than soybean meal (0.69 %:0.68 %) [71]. Accordingly, utilization of canola meal has potential to meet sulfur amino acid requirement for duck as an alternative feed ingredient to soybean meal. Amino acid digestibility of canola meal in duck is presented in Table 8. Lower amino acids digestibility of canola meal than those in soy bean meal except for cysteine was observed in the study with white Pekin ducks [47]. However, this low amino acid digestibility results from higher levels of hulls and tannins in canola compared soybean meal [72]. Nutritional value of canola meals from new varieties of canola when compared to conventional canola meal samples and soybean meals fed to chickens were evaluated [71]. In this study, genetically selected new varieties had more amino acids content and amino acid digestibility with compared to conventional canola meal. It has been concluded that same amino acid digestibility values could be used for ducklings and chicks based on the digestibility studies done with domestic chicks and Muscovy ducks [73].

**Table 8:** Digestibility coefficients of amino acids for duck (3 weeks old white Pekin ducks)

Amino acid	Apparent ileal digestibility (%)		
	[23]	[47]	[74]
Alanine	66.0	79.2	79.3
Arginine	71.0	87.1	87.1
Aspartate + asparagine	60.0	74.6	74.6
Cystine	67.0	70.9	70.9
Glutamate + glutamine	81.0	85.9	85.9
Glycine	59.0	74.5	74.5
Histidine	–	82.7	82.7
Isoleucine	65.0	77.7	77.7
Leucine	73.0	79.4	79.4
Lysine	66.0	79.0	79.0
Methionine	80.0	84.8	84.7
Phenylalanine	73.0	81.5	81.5
Proline	–	75.7	75.7
Serine	70.0	71.4	71.4
Threonine	64.0	69.6	69.6
Tryptophan	–	84.9	84.9
Tyrosine	–	76.4	76.4
Valine	62.0	74.1	74.1

Dashes indicate that no data were available

### Minerals for ducks

The most important minerals for ducks are calcium (Ca) and phosphorus (P). These are needed for bone formation, egg shell formation and maintenance of ducks. Ducklings’ requirement of Ca:P ratio is between 1:1 and 2:1. For laying ducks, this ratio is 6:1 and they need 4.0 g of calcium every day for egg shell formation [75]*.* Furthermore, dietary available phosphorus (AP) levels influenced the mean egg weight and 4.0 % calcium with 0.6 % AP was optimum for indigenous layer ducks [76]*.* It was reported that maximum weight and feed conversion were obtained for White Pekin ducklings when fed practical type rations containing 0.7–0.9 % total phosphorus [77]. Canola meal is enriched with AP (0.38 %) with compared to other vegetable sources like soybean meal (0.28 %) and wheat (0.09 %) [17].

Information on the mineral value of canola meal in duck diets is not accessible. There was no significant effect of replacing soybean meal with canola meal in broiler diet (21 days old broilers) on apparent retention and bone content of calcium and phosphorus [78]. In the same study, no effect of replacing soybean meal with canola meal was observed in laying hen with respect to apparent retention and bone content of calcium and phosphorus [78].

## Feeding value of canola meal for duck

Canola meal is used as alternative protein source in all types of poultry feeds. Last three decades, many studies focused on canola meal utilization on chickens and swine as these are leading livestock industries. Therefore, limited information is available for its utilization for ducks. Canola meal is commonly fed to ducks and geese without any problems [23]. Geese have a greater digestive capability than other types of poultry and hence, it appears to digest canola meal more efficiently than other poultry [79]. It is estimated that up to 15 % of canola meal can be included in a diet for duck without compromising production indices [23, 44]. For broilers (first 5 weeks of age), supplementation of canola meal at the level of 25 % showed maximum weight and improved feed conversion ratio [80]. Conventional canola meal (rapeseed meal with high glucosinolates) showed negative effect on the growth performance of Mule ducks at the starting period [52]. Even though glucosinolates have affected the thyroid functioning and probably the thyroxin production, duck appeared less sensitive and did not adversely affect the meat production during the growing period, and thereby it is possible to use higher incorporation rate in the latter period. Moreover, as canola meal contains low amount of glucosinolates than rapeseed meal aforementioned problems may not exist with canola meal.

Low glucosinolate canola meal (BC 86–16) showed high rate of egg production when supplemented 24–25 % of the diet [81]. Similarly, it was observed no effects on egg production, egg weight and body weights of the layers when soybean meal was partially replaced with canola meal [82].

No adverse sensory effects were observed with canola meal containing diets up to 210 g/kg in the starter with 90 g/kg in the finisher on meat quality of broiler chickens [83]. Furthermore, carcass yields of broiler chickens were not different in broiler chickens fed diets containing either soybean meal or 10 or 20 % full fat canola [84]. In this regard, inclusion of canola meal in duck diets may not affect in meat quality. Nevertheless, canola seed hulls tend to stick in the digestive track of poultry which may lower the carcass quality during processing [23].

## Canola meal enhancement on duck

Canola meal component which impacts on duck health should be considered when feeding. Basically, ANFs play a major role in duck health. As mentioned before in previous section fiber portion of canola meal considered as an ANF. However, cell wall polysaccharides can be converted to the substances that are stimulating healthy microbial growth in the gastrointestinal tract using commercially available in-feed enzymes [85]. Galacto-, gluco-, manno-, or xylo-oligomers can be produced by supplementing non-starch polysaccharide hydrolyzing enzymes in a diet containing canola meal [86], lending prebiotic effect. Prebiotics may selectively encourage favorable microbial population and thereby reduce incidence of enteric pathogens in the intestinal system [87]. When canola meal is treated with carbohydrase enzymes it can reduce substrate availability for harmful microbial growth in the ileum and improve nutrient digestion and absorption [88, 89]. Similarly, multi-carbohydrase enzyme acts on the NSP in canola meal, resulted in reduced amounts of water insoluble NSP and increased amounts of water soluble NSP, and increased NSP hydrolysis products with some monosaccharides. Galactose, glucose and uronic acid were predominant among the released monosaccharides [90]. Similarly, it was documented that correct blend of carbohydrase enzymes acting on NSP could produce low-molecular weight polysaccharides, simple sugars and oligosaccharides, which improve gut environment by being utilized as prebiotics for beneficial microbes in the intestinal tract [91]. Moreover, exogenous enzymes improve digestibility and growth performance of the birds by means of improving gut morphology [92].

## Conclusion

As an alternative feed ingredient, canola is the possible alternative vegetable protein source which can substitute soybean meal. It consists of well-balanced amino acid profile and rich in methionine and cysteine. It also riches in vitamins with compared to other vegetable protein sources. Carcass yield and sensory quality were not affected by feeding canola meal. Downgrading factors like fiber content and phytic acid can be eliminate by using hydrolyzing enzymes. With these enzymes, feeding value of canola meal can be enhanced with potential health benefits in birds. Collectively, it is expected that canola meal shows a great potential for duck nutrition as an alternative feed ingredient. However, more researches are needed to confirm duck performance with canola meal in future.
