# Erratum to: A review of canola meal as an alternative feed ingredient for ducks

**DOI:** 10.1186/s40781-015-0071-3

**Published:** 2015-10-12

**Authors:** Samiru Sudharaka Wickramasuriya, Young-Joo Yi, Jae Cheol Kim, Jaehong Yoo, Nam Kyu Kang, Jung Min Heo

**Affiliations:** Division of Animal and Dairy Science, Chungnam National University, Daejeon, 305-764 Republic of Korea; Division of Biotechnology, College of Environmental & Bioresources, Chonbuk National University, Iksan-si 570-752, Jeollabuk-do, Republic of Korea; Pork Innovation, Department of Agriculture and Food, South Perth, WA 6151 Australia

## Erratum

An author was omitted from the original publication [1]. Jae Cheol Kim has been added as the third author. The author list has been rectified by this Erratum, as has the Authors' contributions section below.
